# The impact of legislative changes on abortion care: A retrospective study of one hospital system in North Carolina

**DOI:** 10.1016/j.srhc.2026.101207

**Published:** 2026-03-15

**Authors:** Morgan E. Johnson, Jessica E. Morse, Amy Bryant

**Affiliations:** a University of North Carolina School of Medicine, 150 Medical Drive, Chapel Hill, NC 27514, USA; b UNC Department of Obstetrics & Gynecology, University of North Carolina School of Medicine, Campus Box 7570, Chapel Hill, NC 27599-7570, USA

**Keywords:** Access to abortion, Abortion legislation, Public health, Abortion-related morbidity and mortality

## Abstract

**Objectives:**

North Carolina is unique as a southeastern state that provides abortion access for the region and implemented a 12 week ban one year after the Dobbs decision. This study sought to compare demographics, gestational duration (GD), and clinical characteristics of abortion patients across three time frames corresponding to federal and state-level policy changes. We compared in-state to out-of-state patients at one North Carolina hospital system between July 2021 and June 2024.

**Study Design:**

We conducted a quasi-experimental retrospective cohort study using electronic health record data, comparing patient characteristics and clinical outcomes across three distinct time periods. Analyses were stratified by residency (in-state versus out-of-state) to evaluate differential impacts over time. Patients were grouped by date: the year before Dobbs (July 2021-June 2022), the year after Dobbs (July 2022-June 2023), and the year after SB20 (July 2023-June 2024). We used descriptive statistics and regression models.

**Results:**

Of 929 patients, 291 were pre-Dobbs, 306 were post-Dobbs, and 333 were post-SB20 with average GDs of 14.9, 13.5, and 9.7 weeks, respectively. Out-of-state patients were 1.6 (OR = 1.57, 95% CI 1.04–2.39) times as likely to have a risk factor for an abortion complication. Time frame and state of residence were significant predictors of GD (*p* < 0.001) but did not predict complications.

**Conclusions:**

Despite a 12 week abortion ban in North Carolina, out-of-state patients continued to seek care at a significantly higher GD which is associated with a higher risk of complications in prior literature.

## Introduction

Induced abortion is a critical component of comprehensive reproductive healthcare. Access to this common reproductive health care is a growing concern for patients, as recent legislation has significantly increased barriers. On June 24, 2022, the US Supreme Court delivered the *Dobbs v Jackson Women’s Health Organization* (Dobbs) decision, holding that there is no constitutional right to abortion [1]. Since Dobbs, 12 states have banned abortion, and 6 states have enforced early gestational duration limits, ranging between 6 and 12 weeks [2,3]. State abortion bans enacted in the two years since Dobbs have left residents of nearly a quarter of US counties having to travel more than 200 miles to find an abortion provider [4], often traveling out-of-state to access care. An estimated 155,000 patients traveled out-of-state for abortion in 2024 [2].

After the Dobbs decision in June 2022, North Carolina was one of the few states in the Southeast continuing to offer care through the second trimester and saw a steep increase in the number of abortion patients from out of state [2]. In August 2022, a 20-week abortion ban that had been enjoined went back into effect in North Carolina [5]. Despite this 20-week limitation, North Carolina remained a significant access state for second trimester abortion, with an average 27% increase in the number of abortions performed monthly during the year post-Dobbs [6]. Before Dobbs, the median daily number of out-of-state abortions in North Carolina was 16.5; during the first year post-Dobbs, the number rose to 55.1, with a total of 17,977 out-of-state abortions occurring that year [6].

Access to abortion changed dramatically again in North Carolina on July 1, 2023 when NC Senate Bill 20 (SB20) went into effect, restricting abortion beyond 12 weeks except in the cases of rape, incest, a “life-limiting fetal anomaly,” or a medical emergency [7]. SB20 also imposed a 72-h in-person waiting period, resulting in the need for at least 2 clinic visits to access an abortion.

These significant changes in abortion policy in North Carolina and the Southeast raise questions about how patient care was impacted. We sought to describe abortion care at an institution in North Carolina to offer insight into the effects of abortion legislation both on the national and state-level.

Although complications from induced abortion at any gestational age are rare, increased GD at the time of abortion does increase the risk of major complications [8,9]. Greater distance from an abortion facility is associated with delays in abortion and increased GD at time of procedure [10]. Anecdotally at our institution, providers noticed higher clinical complexity and acuity among out-of-state abortion patients. This observation led us to question whether out-of-state patients had more risk factors than in-state patients and were at higher risk of complications from abortion when they presented to our institution.

This study aims to compare the differences in patient populations and clinical outcomes at a single academic medical center across three legislative time periods, looking specifically at factors that may have public health implications resulting from changes in federal and state-level policy. The objective of this study was to evaluate differences in patient characteristics, gestational duration, and complication risk across three legislative cohorts, stratified by state of residence. We hypothesized that we would see differences in the populations of patients seeking abortion in these three time periods including more out-of-state patients after Dobbs, and that these patients would present at higher GDs, have more risk factors for complications, and experience more complications. Understanding the differences in GD and proximity to clinic could highlight changes in care after the implementation of restrictions and inform both state and national-level abortion policy.

## Materials and methods

### Data

We conducted a quasi-experimental retrospective cohort study using electronic health record data to compare patient characteristics and clinical outcomes across three distinct time periods corresponding to changes in federal and state-level abortion policy. Each individual contributed data to a single time period only. We further stratified analyses by residency status (in-state vs out-of-state) to assess differential impacts of policy change on these same patient characteristics and clinical outcomes. We reviewed charts of patients seeking induced abortion care at a single institution from July 2021-June 2024. Patients were identified using CPT and ICD-10 codes 59840, 59841, Z33.2, and O03.9. Spontaneous abortions were excluded. We subsequently grouped patients into three distinct time periods: the year prior to Dobbs (July 2021-June 2022),^1^ the year after Dobbs and prior to SB20 (July 2022-June 2023),^2^ and the year after SB20 (July 2023-June 2024).^3^

We collected the following demographic, obstetrical, and medical history: age, race and ethnicity, zip code of primary residence, insurance status, gravidity, parity, pregnancy history, mode of delivery, past pregnancy complications (preeclampsia, gestational hypertension, HELLP, presence of placenta accreta spectrum, PPROM, preterm birth, and cervical insufficiency), and past medical history (coagulopathies, hypertension, cardiac disease, stroke, diabetes, and presence of fibroids). We also collected abortion specific information including method of abortion (medication, vacuum aspiration, dilation and evacuation), GD at time of abortion, starting hemoglobin, and any complications from the abortion.

We defined risk factors for complication from abortion as previous cesarean section, known coagulopathy, GD ≥ 20 weeks, prior post-partum hemorrhage, prior stroke, starting haemoglobin <10 mg/dL (defined as moderate or severe anemia in pregnancy), placenta accreta spectrum, and fibroids [11–17].

To comprehensively track all abortion-associated complications, we reviewed medical records up to three months following the abortion. We defined major complications as hemorrhage requiring transfusion, infection requiring intravenous administration of antibiotics, uterine perforation requiring hospitalization or surgical intervention, abdominal surgical procedure (hysterectomy, laparotomy), abortion-related hospitalization, and abortion-related death [18]. We defined minor complications as excessive blood loss not requiring transfusion, infection requiring outpatient treatment with antibiotics, minor trauma to the cervix or vagina requiring surgical repair and repeat aspiration [18]. We defined excessive blood loss as bleeding over 500 mLs and necessitating 1 or more of the following: 3 or more uterotonic medication doses, placement of an intrauterine balloon catheter, re-aspiration, or additional interventions [19]. Ethical approval was received from the Biomedical Institutional Review Board.

### Statistical analysis

Demographics were summarized using descriptive statistics. To assess for any differences in patient populations between the time periods, we used chi-square for categorical variables including patient age, race or ethnicity, insurance status, North Carolina residency, gravidity, parity, and type of abortion. A multivariate linear regression model was used to evaluate associations between GD and either time frame or state of residency. A series of binomial logistic regression models were used to evaluate associations between proposed predictors (i.e., state of residence and time frame) and clinical outcomes (i.e., complications and risk factors).

## Results

### Patient characteristics by time frame

Between July 2021 and June 2024, we identified 929 abortions, including 290 (31.3%) pre-Dobbs, 306 (32.9%) post-Dobbs, and 333 (35.8%) post-SB20 (Table 1). Across the three time frames, patient age distribution, race and ethnicity, gravidity, parity, and insurance status did not differ significantly. In contrast, state of residence varied significantly by time frame. While 88.2% of all patients were North Carolina residents, the proportion of out-of-state patients increased from 7.9% pre-Dobbs to 22.1% post-Dobbs, then declined to 6.0% post-SB20 (p < 0.001). Abortion type also differed significantly across time frames (p < 0.001). Medication abortions increased over the three time periods, from 9.7% pre-Dobbs to 17.0% post-Dobbs, to 48.3% post-SB20. Dilation and evacuation (D&E) procedures decreased from 57.9% pre-Dobbs to 44.8% post-Dobbs and 15.3% post-SB20. Gestational duration (GD) likewise shifted across periods. Mean GD decreased from 14.9 weeks pre-Dobbs to 13.5 weeks post-Dobbs, to 9.7 weeks post-SB20.

### Patient characteristics by state of residence across time frames

When stratified by state of residence, age, race and ethnicity, gravidity, and parity were similar between in-state and out-of-state patients across all time frames (Table 2). However, insurance status differed significantly by state of residence. Compared with in-state patients, out-of-state patients were more likely to be uninsured and less likely to have Medicaid or Medicare coverage (p < 0.001). These insurance differences were consistent across time frames. Abortion type also differed substantially by residence status. Medication abortion was more common among in-state patients (28.7%) than out-of-state patients (5.5%), whereas D&E procedures predominated among out-of-state patients (61.8%) compared with in-state patients (35.2%) (p < 0.001). These differences persisted across all three time frames. Out-of-state patients presented at later gestational durations than in-state patients in each period.

### Risk factors for abortion complications by time frame

Overall, 46.1% of patients had at least one risk factor for abortion complications (Table 1). The most common risk factors were prior cesarean delivery (21.7%), gestational duration ≥ 20 weeks (16.9%), history of uterine fibroids (8.9%), and hemoglobin < 10 g/dL (7.8%) (Data not shown). The proportion of patients with any risk factor declined over time, from 58.6% pre-Dobbs to 44.1% post-Dobbs and 38.4% post-SB20. This decline mirrored reductions in later gestational duration and D&E procedures over time.

### Risk factors for abortion complications by state of residence

Across time frames, out-of-state patients were more likely to have at least one risk factor for abortion complications than in-state patients. This difference was most pronounced during the post-Dobbs period, when 78.3% of out-of-state patients had at least one risk factor compared with 56.9% of in-state patients (Table 2). These differences paralleled later gestational duration and higher proportions of D&E procedures among out-of-state patients in each time frame.

### Complications by time frame and state of residence

Overall, 7.8% of abortions resulted in a complication, including 1.6% major complications and 6.2% minor complications (Table 1). Complication rates did not differ significantly across time frames.

The most common major complications were hemorrhage requiring transfusion (1.1%) and abortion-related hospitalization (0.9%). The most common minor complications included repeat aspiration (2.7%), uterine atony (2.1%), and hemorrhage not requiring transfusion (1.9%) (Data not shown). When stratified by state of residence, complication rates were similar between in-state and out-of-state patients across all time frames, despite differences in gestational duration and procedure type.

### Odds ratios for risk factors and complications

Using binomial logistic regression models, time frame, state of residence, and type of abortion were independently associated with the presence of any risk factor, but not with the occurrence of complications. Compared with pre-Dobbs patients, those in the post-Dobbs period were 48% less likely to have a risk factor (OR 0.52, 95% CI 0.37–0.73), and those post-SB20 were 56% less likely (OR 0.44, 95% CI 0.32–0.61) (Table 3). Out-of-state patients had higher odds of having a risk factor compared with in-state patients (OR 1.58, 95% CI 1.04–2.39). Compared to patients who had medication abortions, those who underwent uterine aspiration (OR 2.15, 95% CI 1.47–3.16) and D&E (OR 6.44, 95% 4.25–9.75) were more likely to have a risk factor.

Neither time frame, state of residence, nor type of abortion significantly predicted any complication, major complication, or minor complication.

### Regression analysis of gestational duration

Using a multivariable linear regression model, time frame and state of residence independently predicted gestational duration at presentation. Together, these variables explained approximately 18.0% of the variance in gestational duration (adjusted R^2^ = 0.180; F[2926] = 102.9, p < 0.001). Both predictors were statistically significant (p < 0.001), with later policy periods and out-of-state residence associated with increased gestational duration (Table 4).

## Discussion

Accessing abortion care has changed significantly due to the Dobbs decision and subsequent state legislation on abortion, particularly in the southeastern United States. With this study we sought to evaluate changes in demographics and clinical characteristics of patients receiving abortion care at a single hospital system within North Carolina between July 2021, the year prior to Dobbs, and July 2024, the year after restrictive legislation in North Carolina (SB20) was passed. This quasi-experimental retrospective cohort design allowed us to examine how varying federal and state policies across time frames impacted patient characteristics and outcomes. We did not find a straightforward relationship between being out-of-state and having a higher complication rate. Patients from out of state were not more likely to have complications, but they were more likely to have higher gestational durations and risk factors for abortion complications both in the post-Dobbs and the post-SB20 time frames. This seemingly inconsistent finding may be reflective of the known safety and low complication rates associated with abortion, even in the presence of known risk factors.

Interestingly, despite the introduction of restrictive abortion legislation, the number of patients increased across time frames. The post-Dobbs time frame saw state-level abortion restriction changes in South Carolina, Tennessee, Georgia, Alabama and Mississippi. The increase in abortions in North Carolina post-Dobbs is reflected both in state-level data and at our institution [6]. This suggests that abortion care continues to be pursued, despite barriers such as interstate travel and gestational age limits. This trend persisted even after the introduction of SB20. In North Carolina, 50,980 abortions were performed in the year post-Dobbs, and 53,650 abortions in the year post-SB20. This is in stark contrast to 2022 and 2021 when 39,286 [8] and 32,454 [20] abortions were performed.

We found a statistically significant difference between in-state and out-of-state patients in regards to type of abortion. Overall, a greater proportion of out-of-state patients had procedural abortions. Additionally, while the overall GD decreased across time frames, out-of-state patients continued to have relatively higher GD compared to their in-state counterparts. Our findings are consistent with other studies documenting higher gestational ages for patients seeking care out of state [21,22]. Due to the referral nature of much of the care provided at our institution, which is equipped to provide care to the most acute and complex patients, abortion type skews towards procedural abortion. Telemedicine for medication abortion was not legal in North Carolina during the time frames of the study, which may also explain why more procedural care was provided [23]. Furthermore, our data does not capture other clinical settings where a commensurate increase in medication abortions may have occurred. While we were unable to assess how far individual patients traveled to receive care and the specific barriers they may have faced, current literature strongly suggests greater distance is intrinsically linked to delays in care resulting in presenting at a higher GD, which could preclude eligibility for a medication abortion [10,24–27].

Following the Dobbs decision, the proportion of out-of-state patients significantly increased from 7.9% to 22.1%. This is likely reflective of North Carolina being one of the only southeastern states to allow abortions after the first trimester post-Dobbs. For the first two months after Dobbs, North Carolina providers were able to perform abortions through viability for any indication. In August 2022, a 20-week ban went back into effect, limiting but not eliminating access to second trimester abortion in North Carolina [5]. North Carolina remained a critical state for second trimester abortion access in the year post-Dobbs. Similar trends were seen in other states without pre-viability restrictions in the year following Dobbs [2,21,28]. A study in Washington state found that more out-of-state patients were accessing abortions following Dobbs [22]. As would be expected, after implementation of a 12-week ban with limited exceptions and a 72-h in-person waiting period, the percentage of patients from out-of-state decreased below pre-Dobbs levels; however the mean gestational duration was still increased relative to in-state patients, suggesting that North Carolina was still a point of access for patients from more restrictive states meeting exception criteria for abortions over 12 weeks.

Our study also sought to evaluate whether patients traveling from out of state would present with more risk factors for abortion complications and experience higher complication rates than in-state patients. Complication rates vary and depend on procedure type, GD, patient comorbidities, and clinician experience [29,30]. Current literature suggests that the total abortion-related major complication rate is estimated to be about 2%, with the incidence of major complications <0.2% and minor complications <4% [30–32]. Our study found an overall rate of any complication to be 7.8% (1.6% major, 6.2% minor). We attribute this higher than anticipated complication rate to our hospital system’s role as a high acuity tertiary care center that provides care to individuals with multiple comorbidities often necessitating hospital-based care and presenting at higher GDs. We were unable to assess for differences in major complications across time frame or by state of residence due to low absolute numbers. However, considering out-of-state patients were 1.6 times likelier to have at least one risk factor, undergo D&E, and had significantly higher GDs, these individuals could have been at higher risk of complications, even though we did not find state of residence, type of abortion, or time frame to predict having a complication.

Our study has several limitations. While we reviewed medical records for complications, it is possible that we are missing data on complications for those who do not routinely seek care at this hospital system or in North Carolina. Additionally, this hospital system does not represent most abortion care, which is done in high volume, low acuity centers with low rates of major and minor complications. Although it is critical for patients with higher risk clinical situations to have access to a tertiary care hospital system like the one described in this study, those patients are not representative of abortion patients nationally who tend to be young and healthy. However, this study captures higher-acuity abortion care not otherwise reflected in clinic-based datasets and therefore provides insight into a clinically complex subset of patients that otherwise may be absent from much of the existing literature. Additionally, despite this study describing a higher acuity hospital system, the low number of major and minor complications limits statistical power. We did not assess precise distance traveled to receive care; however the site of this study is a North Carolina hospital in the center of the state, allowing us to assume out-of-state residents from neighboring states are traveling at minimum 124 miles from the South Carolina border, 286 miles from the Georgia border, and 177 miles from the Tennessee border. Due to the retrospective nature of our data collection, we were not able to assess patients for potential barriers faced in accessing care. We were unable to draw any conclusions about out-of-state patients and economic barriers to abortion. We attempted to use insurance status as a proxy for socioeconomic status (SES). However, no state Medicaid in the Southeast covers abortion except in the federally indicated circumstances and many private insurers across the Southeast also do not cover abortion. Out-of-state patients pay out-of-pocket and do not have their actual insurance status documented in our medical records. Thus, the differences we found in insurance status between in-state and out-of-state patients are difficult to interpret and may be more reflective of abortion coverage differences rather than actual insurance status or SES.

This study provides evidence that out-of-state patients continue to present at significantly higher gestational durations than their in-state counterparts despite overall average GD decreasing significantly across time frames as abortion restrictions increased in North Carolina. Our study did not find that being an out-of-state patient was associated with a higher rate of complications. Higher GDs alone put these patients at increased risk for complications for a procedure that could be done earlier in the patient’s home state, were it not for restrictions [8,9].

## Implications for practice and/or policy

This study describes patients seeking abortion at one hospital system in North Carolina between July 2021 and June 2024 across three different legislative time periods. Out-of-state patients presented at significantly higher gestational durations and with more risk factors for complications. This suggests barriers to abortion care, including legal restrictions, have the potential to negatively impact abortion-related morbidity and mortality.

Federal and state-level changes in abortion policy are complex and overlapping, complicating the delivery and receipt of care beyond state borders. Our findings provide a unique picture of how abortion care changed at a tertiary care hospital over the time frames of three significant federal and state-level policy changes. These findings can inform state policies and practices to improve access for all seeking abortion care.

## Figures and Tables

**Table 1:** Characteristics of Patients Seeking Abortion Between July 2021 – June 2024.

Characteristics	Total Patients N = 929 (%)	Pre-Dobbs n = 290 (%)	Post-Dobbs n = 306 (%)	Post-SB20 n = 333	*P* Value
**Age (y)**					0.28
<15	11 (1.2)	4 (1.4)	5 (1.6)	2 (0.6)	
15–19	63 (6.8)	23 (7.9)	19 (6.2)	21 (6.3)	
20–24	150 (16.2)	32 (11.0)	49 (16.0)	69 (20.7)	
25–29	243 (26.2)	74 (25.5)	85 (27.8)	83 (24.9)	
30–34	219 (23.6)	73 (25.2)	68 (22.2)	78 (23.4)	
35–39	173 (18.6)	59 (20.3)	59 (19.3)	56 (16.8)	
40 or older	70 (7.5)	25 (8.6)	21 (6.9)	24 (7.2)	
**Race or ethnicity**					0.24
Non-Hispanic white	340 (36.6)	106 (36.9)	123 (40.2)	111 (33.3)	
Non-Hispanic Black	359 (38.6)	114 (39.5)	119 (38.9)	126 (37.8)	
Hispanic	124 (13.4)	32 (11.0)	35 (11.4)	57 (17.1)	
Asian	51 (5.5)	18 (6.2)	12 (3.9)	21 (6.3)	
Other^a^	55 (5.9)	20 (6.9)	17 (5.6)	18 (5.4)	
**Insurance Status**					0.86
Private Insurance	473 (51.0)	147 (50.7)	160 (52.3)	166 (49.8)	
Tricare	35 (3.8)	12 (4.1)	12 (3.9)	11 (3.3)	
Medicaid/Medicare	338 (36.4)	108 (37.2)	105 (34.3)	125 (37.5)	
None	72 (7.8)	22 (7.6)	25 (8.2)	25 (7.5)	
Unknown	11 (1.2)	1 (0.3)	4 (1.3)	6 (1.8)	
**North Carolina Resident**					<0.001
Yes	819 (88.2)	267 (92.1)	239 (78.1)	313 (94.0)	
No	110 (11.8)	23 (7.9)	67 (21.9)	20 (6.0)	
**Gravidity**					0.40
1	258 (27.8)	74 (25.5)	97 (31.7)	87 (26.1)	
2	185 (19.9)	62 (21.4)	55 (18.0)	68 (20.4)	
3	188 (20.2)	65 (22.4)	46 (15.0)	77 (23.1)	
4	113 (12.2)	37 (12.8)	44 (14.4)	32 (9.6)	
5 or more	185 (19.9)	52 (17.9)	65 (21.2)	69 (20.7)	
**Parity**					0.35
0	337 (36.3)	96 (33.1)	117 (38.2)	124 (37.2)	
1	246 (26.5)	76 (26.1)	81 (26.5)	89 (26.7)	
2	193 (20.8)	70 (24.1)	55 (18.0)	68 (20.4)	
3	79 (8.5)	30 (10.3)	25 (8.2)	24 (7.2)	
4 or more	74 (8.0)	18 (6.2)	29 (9.5)	28 (8.4)	
**Type of Abortion**					<0.001
Medication	241 (25.9)	28 (9.7)	52 (17.0)	161 (48.3)	
Uterine Aspiration	331 (35.6)	93 (32.1)	117 (38.2)	121 (36.3)	
D&E	356 (38.3)	168 (57.9)	137 (44.8)	51 (15.3)	
**GD (weeks)**					<0.001
≤6w6d	168 (18.1)	28 (9.7)	34 (11.1)	106 (33.8)	
7w0d–12w6d	322 (34.7)	69 (23.8)	91 (29.7)	162 (48.7)	
13w0d–20w6d	346 (37.2)	134 (46.0)	168 (54.9)	44 (13.2)	
≥21w0d	93 (10)	59 (20.3)	13 (4.3)	21 (6.3)	
**Avg. GD** (SD)	12.5 (5.63)	14.9 (5.6)	13.5 (5.1)	9.7 (4.9)	<0.001
**Any risk factor(% Yes)**	433 (46.1)	170 (58.6)	135 (44.1)	128 (38.4)	<0.001
**Any complication(% Yes)**	72 (7.8)	23 (7.9)	29 (9.5)	20 (6.0)	0.31

Table 1 describes the patient demographics, obstetric histories, and abortion information of all patients seeking abortion by time frame.

a Other includes Alaskan, American Indian, Hawaiian, Samoan, unknown, or other race or ethnicity.

**Table 2:** Characteristics of Patients Seeking Abortion Between July 2021 – June 2024 by in-state versus out-of-state status.

Characteristics	Total Patients N = 929			Pre-Dobbs		Post-Dobbs		Post-SB20		
				n = 290		n = 306		n = 333		
	In-state	Out-of-state	*P* Value	In-state	Out-of-state	In-state	Out-of-state	In-state	Out-of-state	*P* Value
	n = 819 (%)	n = 110 (%)		n = 267 (%)	n = 23 (%)	n = 239 (%)	n = 67 (%)	n = 313 (%)	n = 20 (%)	
**Age (y)**			0.183							0.28
<15	9 (1.1)	2 (1.8)		4 (1.5)	0	3 (1.2)	2 (3)	2 (0.6)	0	
15–19	58 (7.1)	5 (4.6)		22 (8.2)	1 (4.3)	16 (6.7)	3 (4.5)	20 (6.4)	1 (5)	
20–24	136 (16.6)	14 (12.7)		28 (10.5)	4 (17.4)	42 (17.6)	7 (6.4)	66 (21.1)	3 (15)	
25–29	209 (25.5)	34 (30.9)		66 (24.7)	8 (34.8)	65 (27.2)	21 (31.3)	78 (24.9)	5 (25)	
30–34	193 (23.6)	26 (23.6)		66 (24.7)	7 (30.4)	52 (21.8)	16 (23.9)	75 (24)	3 (15)	
35–39	147 (18)	26 (23.6)		56 (20.8)	3 (13.1)	43 (18)	15 (22.4)	48 (15.3)	8 (40)	
40 or older	67 (8.2)	3 (2.7)		25 (9.4)	0	18 (7.5)	3 (4.5)	24 (7.7)	0	
**Race or ethnicity**			0.307							0.24
Non-Hispanic white	298 (36.4)	42 (38.2)		98 (36.7)	8 (34.8)	95 (39.7)	28 (41.7)	105 (33.5)	6 (30)	
Non-Hispanic Black	311 (38)	48 (43.6)		102 (38.2)	12 (52.2)	93 (38.9)	26 (38.8)	116 (37.1)	10 (50)	
Hispanic	110 (13.4)	14 (12.7)		31 (11.6)	1 (4.3)	26 (10.9)	9 (23.4)	53 (16.9)	4 (20)	
Asian	48 (5.9)	3 (2.7)		17 (6.3)	1 (4.3)	10 (4.2)	2 (3)	21 (6.7)	0	
Other^1^	52 (6.4)	3 (2.7)		19 (7.1)	1 (4.3)	15 (6.3)	2 (3)	18 (5.8)	0	
**Insurance Status**			<0.001							0.86
Private	409 (49.9)	64 (58.2)		137 (51.3)	10 (43.5)	116 (48.5)	44 (65.7)	156 (49.8)	10 (50)	
Tricare	32 (3.9)	3 (3.7)		12 (4.5)	0	10 (4.2)	2 (3)	10 (32)	1 (5)	
Medicaid/Medicare	319 (39)	19 (17.3)		102 (38.2)	6 (26.1)	96 (40.2)	9 (13.4)	121 (38.7)	4 (20)	
None	50 (6.1)	22 (20)		15 (5.6)	7 (30.4)	14 (5.9)	11 (16.4)	21 (6.7)	4 (20)	
Unknown	9 (1.1)	2 (1.8)		1 (0.4)	0	3 (1.3)	1 (1.5)	5 (1.6)	1 (5)	
**Gravida**			0.964							0.4
1	223 (27.2)	35 (31.8)		67 (25.1)	7 (30.4)	70 (29.3)	27 (40.3)	86 (27.5)	1 (5)	
2	162 (19.8)	23 (20.9)		60 (22.5)	2 (8.7)	38 (15.9)	17 (25.4)	64 (20.5)	4 (20)	
3	170 (20.8)	18 (16.4)		58 (21.7)	7 (30.4)	40 (16.7)	6 (9)	72 (23)	5 (25)	
4	99 (12.1)	14 (12.7)		33 (12.4)	4 (17.4)	37 (15.5)	7 (10.5)	29 (9.3)	3 (15)	
5 or more	165 (20.1)	20 (18.2)		49 (18.4)	3 (13)	54 (22.6)	10 (14.9)	62 (19.8)	7 (35)	
**Parity**			0.88							0.35
0	292 (35.7)	45 (40.9)		87 (32.6)	9 (39.1)	84 (35.2)	33 (49.3)	121 (38.7)	3 (15)	
1	221 (27)	25 (22.7)		74 (27.7)	2 (8.7)	65 (27.2)	16 (23.9)	82 (26.2)	7 (35)	
2	173 (21.1)	20 (18.2)		64 (24)	6 (26.1)	44 (18.4)	11 (16.4)	65 (20.8)	3 (15)	
3	69 (8.4)	10 (9.1)		26 (9.7)	4 (17.4)	22 (9.2)	3 (4.5)	21 (6.7)	3 (15)	
4 or more	64 (7.8)	10 (9.1)		16 (6.0)	2 (8.7)	24 (10)	4 (6)	24 (7.7)	4 (20)	
**Type of Abortion**			<0.001							<0.001
Medication	235 (28.7)	6 (5.5)		28 (10.5)	0	49 (20.5)	3 (4.5)	158 (50.5)	3 (15)	
Uterine Aspiration	295 (36.0)	36 (32.7)		89 (33.3)	4 (17.4)	96 (40.2)	21 (31.3)	89 (28.4)	11 (55)	
D&E	288 (35.2)	68 (61.8)		149 (55.8)	19 (82.6)	94 (39.3)	43 (64.2)	149 (47.6)	6 (30)	
**GD (weeks)**			<0.001							<0.001
≤6w6d	165 (20.1)	3 (2.7)		27 (10.1)	1 (4.3)	32 (13.4)	2 (3)	106 (33.9)	0	
7w0d–12w6d	296 (36.1)	26 (23.6)		67 (25.1)	2 (8.6)	81 (33.4)	10 (14.9)	148 (47.3)	14 (70)	
13w0d–20w6d	280 (34.2)	66 (60)		124 (46.4)	10 (43.5)	116 (48.5)	52 (77.6)	40 (12.8)	4 (20)	
≥21w0d	78 (9.5)	15 (13.6)		49 (18.4)	10 (43.5)	10 (4.2)	3 (4.5)	19 (6.1)	2 (10)	
**Avg. GD** (SD)	12.12 (5.7)	15.67 (4.4)	<0.001	14.57 (5.6)	18.30 (4.3)	12.87 (5.3)	5.52 (4.0)	9.45 (4.8)	13.15 (4.3)	<0.001
**Any risk factor?(% Yes)**	372 (45.4)	61 (55.5)	0.048	152 (56.9)	18 (78.3)	104 (43.5)	31 (46.3)	116 (37.1)	12 (60)	<0.001
**Any complication?(% Yes)**			0.603							0.53
	61 (7.5)	11 (10)		18 (6.7)	5 (21.7)	24 (10)	5 (7.5)	19 (6.1)	1 (5)	

Table 2 describes the patient demographics, obstetric histories, and abortion information of all patients seeking abortion by time frame and state of residence

**Table 3:** Presence of Risk Factor and Complication.

	Any Risk Factor		Any Complication	
Variable name	Odds ratio	95% CI	Odds ratio	95% CI
**Time Frame**				
Pre-Dobbs (ref)	1.00		1.00	
Post-Dobbs	0.52*	0.37–0.73	1.18	0.66–2.12
Post-SB20	0.44*	0.32–0.61	0.75	0.40–1.39
**State of Residence**				
In-State (ref)	1.00		1.00	
Out-of-State	1.58*	1.04–2.39	1.24	0.62–2.48
**Type of Abortion**				
Medication (ref)	1.00		1.00	
Uterine Aspiration	2.15*	1.47–3.16	0.73	0.34–1.54
D&E	6.44*	4.25–9.75	1.84	0.92–3.71

(ref) = reference group.

Table 3 displays the results of binominal logistic regression models analyzing the presence of any risk factor or any complication by time frame, state of residence, or type of abortion.

* P < 0.05.

**Table 4:** Multivariate Linear Regression Analysis of Gestational Duration (weeks).

Predictor	β	95% Confidence Interval	p
**Time Frame**	−0.38	−2.98 −2.18	<0.001
**State of Residence**	−0.19	−4.35 −2.32	<0.001

Table 4 displays the results of a multivariate linear regression model analyzing time frame and state of residence as predictors of gestational duration.

## Footnotes

1 Gestational limit: to viability.

2 Gestational limit: 20-week ban went into effect August 1, 2022.

3 Gestational limit: 12-week ban, 20-week limit for rape and incest, to viability for fetal indications and maternal health.
